# Estimating effective connectivity in Alzheimer's disease progression: A dynamic causal modeling study

**DOI:** 10.3389/fnhum.2022.1060936

**Published:** 2022-12-15

**Authors:** Jiali Huang, Jae-Yoon Jung, Chang S. Nam

**Affiliations:** ^1^Edward P. Fitts Department of Industrial and Systems Engineering, North Carolina State University, Raleigh, NC, United States; ^2^Department of Industrial and Management Systems Engineering, Kyung Hee University, Yongin-si, South Korea; ^3^Department of Big Data Analytics, Kyung Hee University, Yongin-si, South Korea

**Keywords:** Alzheimer's disease (AD), mild cognitive impairment (MCI), effective connectivity, dynamic causal modeling, resting-state fMRI

## Abstract

**Introduction:**

Alzheimer's disease (AD) affects the whole brain from the cellular level to the entire brain network structure. The causal relationship among brain regions concerning the different AD stages is not yet investigated. This study used Dynamic Causal Modeling (DCM) method to assess effective connectivity (EC) and investigate the changes that accompany AD progression.

**Methods:**

We included the resting-state fMRI data of 34 AD patients, 31 late mild cognitive impairment (LMCI) patients, 34 early MCI (EMCI) patients, and 31 cognitive normal (CN) subjects selected from the Alzheimer's Disease Neuroimaging Initiative (ADNI) database. The parametric Empirical Bayes (PEB) method was used to infer the effective connectivities and the corresponding probabilities. A linear regression analysis was carried out to test if the connection strengths could predict subjects' cognitive scores.

**Results:**

The results showed that the connections reduced from full connection in the CN group to no connection in the AD group. Statistical analysis showed the connectivity strengths were lower for later-stage patients. Linear regression analysis showed that the connection strengths were partially predictive of the cognitive scores.

**Discussion:**

Our results demonstrated the dwindling connectivity accompanying AD progression on causal relationships among brain regions and indicated the potential of EC as a loyal biomarker in AD progression.

## 1. Introduction

Alzheimer's disease (AD) is a neurodegenerative disorder that leads to the death of nerve cells and loss of brain tissues, which result in brain shrinkage causing disturbed brain functioning (Deeksha and Abhishek, 2019). There are multiple stages of the disorder: cognitively normal (CN), early mild cognitive impairment (EMCI), late mild cognitive impairment (LMCI), and Alzheimer's disease (AD) (Ramzan et al., 2020). CN subjects age normally with no sign of depression or dementia, while EMCI and LMCI subjects suffer from difficulties in daily life activity caused by the progressed disease. AD is the advanced and final stage of the disease leading to death.

### 1.1. Brain regions and networks associated with AD

Neural imaging analyzes in the past have identified AD-affected brain regions by recording brain activities during both cognitive tasks and resting state. For example, an fMRI study of a learning task found that compared to CN subjects, people with AD exhibit reduced brain activity in the parietal and hippocampal regions during information encoding (Rombouts et al., 2000). This was further confirmed by Smith (2002) in which they found the most severe volume reduction in the hippocampus (HC) important for the formation of new memories. Another important brain region whose reduced activity is associated with AD regardless of subtypes is the posterior cingulate cortex (PCC) (Herholz et al., 2018). PCC is a critical part of the retrieval process of episodic memories (Greicius et al., 2003). Reduced activity in this region is associated with decreased cognitive performance and memory issues.

Bernard et al. (2015) identified PCC as the most connected brain region in the altered brain networks of groups suffering from memory declines. As a vital node in the default mode network (DMN), PCC is connected with the precuneus (Prec), medial prefrontal cortex (mPFC), intraparietal cortex (IPC), inferior temporal cortex (ITC), and HC (Buckner et al., 2008). Those brain regions in DMN tend to be active in a conscious resting state with stimulus-independent thought, representing a default mode of brain function (Greicius et al., 2003). Studies of resting glucose metabolism and brain atrophy showed disruptions in the DMN of AD patients. Similar disruptions were also found in subjects at genetic risk for AD, implying the changes in DMN occur early in the course of the disease (Su et al., 2017).

Other brain networks are also affected by AD progression. Chand et al. (2017b) studied the modulatory interactions between DMN, salience network (SAN), and central executive network (CEN) in subjects with normal cognition and MCI. They found SAN modulates the interaction between the DMN and CEN, and such modulation was disrupted in MCI. CEN, anchored in the dorsolateral prefrontal cortex (dlPFC) and posterior parietal cortex, is widely reported to be more activated for cognitive functions such as attention, working memory, and decision-making (Bor and Seth, 2012). SAN, anchored in the insula and anterior cingulate cortex (ACC), was also studied to understand the altered patterns of cognitive impairment (Chand et al., 2017a). The connections between DMN, SAN, and CEN in AD patients remain an interesting and understudied topic.

While brain regions are considered responsible for specific functions, information is passed around through structural, functional, and effective connections. The temporal correlations among the affected brain regions were studied using functional connectivity (FC). Reduction of FC in the DMN was primarily and consistently reported in AD compared with MCI patients and CN subjects (Greicius et al., 2004; Wu et al., 2011; Grieder et al., 2018; Soman et al., 2020). From the early stages to the late stages of AD, generally reduced correlations within five studied networks including DMN, SAN, dorsal attention network, control network, and sensory-motor network were reported (Brier et al., 2012). The decreased FCs between the posterior part of the cerebral cortex (Prec & PCC) and the anterior parts (ACC & mPFC) were particularly significant in AD patients (Ibrahim et al., 2021).

Representing the directional causal relationships between brain regions, effective connectivity (EC) depicts the influence that one neural system exerts over another (Friston et al., 1993). As opposed to the FC quantified with measures of statistical dependencies, EC corresponds to the parameter of a model that tries to explain the cause of such dependencies (Friston, 2011). Compared to CN subjects, AD patients showed reduced EC within DMN (Zhong et al., 2014). Both the intensity and quantity of the connections decreased and the inter-network interactions were also weaker than that of CN subjects (Liu et al., 2012). Wu et al. (2011) found the ECs from HC to IPC, mPFC, and PCC were all lost in AD patients. ECs in CEN were also disturbed by AD progression. Cai et al. (2017) reported decreased EC within the dlPFC → caudate → thalamus → dlPFC circuit. Using such differences in the EC circuit, their results distinguished MCI patients who since reverted to the normal functioning state, patients who maintained the MCI state, and patients who progressed to AD. Although the small handful of studies shed some light on the connectivity pattern difference between CN subjects and AD patients, no investigation was done on the strengths (i.e., the scaled coupling rate between regions) of such connections. The relationships between the connection strengths and severity of AD were also neglected.

### 1.2. Effective connectivity estimated by dynamic causal modeling

To study the EC of AD patients, we use the dynamic causal modeling method on resting-state fMRI. First introduced in 2003, DCM has quickly become the most popular approach to EC (Friston et al., 2013). DCM regards the propagation of neural activity through brain networks as an input-state-output system (Friston et al., 2003). It infers effective connections under the Bayesian framework to find the model best explaining the observed data. DCM models are motivated by the biophysical behaviors of the neuronal system, thus they reflect empirical knowledge about the connection strength parameters (Stephan et al., 2010).

To infer the connection strengths, Parametric Empirical Bayes (PEB) method is used. This hierarchical method is used to quantify the commonalities and differences across subjects by collating parameters of interest in a two-level model (within-subject level and between-subject level) (Zeidman et al., 2019). The connectivity strengths allow us to into the modulatory effect of the AD progression.

The aim of this study is to investigate the EC between brain regions in different AD progression stages. EC of each subject and group will be estimated using DCM so the directions and intensities of the connections can be compared to reveal the development of AD. We expect that the strength of the connections will be weaker in AD and LMCI subjects compared to EMCI and CN subjects, and that the connectivity strength will be positively related to the mini-mental state examination (MMSE) scores and negatively related to clinical dementia rating (CDR) scores. The worse the cognitive test performance, the weaker the connection.

## 2. Materials and methods

### 2.1. fMRI data

Data used in this study were obtained from the Alzheimer's Disease Neuroimaging Initiative (ADNI) database (adni.loni.usc.edu). The ADNI was launched in 2003 as a public-private partnership, led by Principal Investigator Michael W. Weiner, MD. The primary goal of ADNI has been to test whether serial magnetic resonance imaging (MRI), positron emission tomography (PET), other biological markers, and clinical and neuropsychological assessment can be combined to measure the progression of MCI and early Alzheimer's disease (AD).

Under ADNI, there are numerous research data sets available. We used fMRI data from subjects whose longitudinal records of the visits were fully documented and publicly available. To ensure the uniformity of data acquisition protocols and formats, all images and corresponding clinical data (e.g., mini-mental state examination, [MMSE], clinical dementia rating, [CDR]) were downloaded from ADNI-2 phase since it has most of the fMRI data. We selected four types of subjects, whose general inclusion/exclusion criteria are as follows: (1) CN subjects: MMSE scores above 24, CDR = 0, free of memory complaints; (2) EMCI patients: MMSE scores above 24, CDR = 0.5, have subjective memory complaints, abnormal memory functions; (3) LMCI patients: MMSE scores above 24, CDR = 0.5, have subjective memory complaints, abnormal memory functions (more severe compared to EMCIs); (4) AD patients: MMSE scores ranging from 20 to 26, CDR above 0.5, have subjective memory complaints, abnormal memory functions (same as LMCIs).

In this study, 34 AD patients, 31 LMCI patients, 34 EMCI patients, and 31 CN subjects were selected and analyzed. All subjects remained in their progression stage (stable) throughout the entire data collection visits (spanning for at least 24 months). The average age was 76.65, 72.00, 73.00, and 73.34 for CNs, EMCIs, LMCIs, and ADs, respectively. Details of the demographic and clinical information could be found in Table 1.

**Table 1:** Demographics and clinical information.

	**CN**	**EMCI**	**LMCI**	**AD**	***p*-value**
Gender	19F/12M	24F/10M	13F/18M	18F/16M	
Age	76.65 ± 6.40	72.00 ± 6.56	73.00 ± 8.51	73.34 ± 7.36	0.059
MMSE score	29.32 ± 0.93	27.91 ± 1.89	27.54 ± 1.87	19.80 ± 4.02	<0.001
CDR score	0.00 ± 0.00	0.47 ± 0.12	0.48 ± 0.10	1.02 ± 0.41	<0.001

### 2.2. Data acquisition and preprocessing

The data were acquired on a 3.0-T (Philips) scanner with TR/TE set as 3,000/30 ms and flip angle of 80. Resting-state functional images were obtained using an echo-planar imaging sequence (EPI). Each series has 140 volumes, and each volume consists of 48 slices of image matrices with dimensions 64 × 64 with voxel size of 3.31 × 3.31 × 3.31 *mm*^3^. During the fMRI scans, all participants were instructed to keep their eyes open and relax.

The preprocessing was carried out using Statistical Parametric Mapping 12 (SPM 12, http://www.fil.ion.ucl.ac.uk/spm) and RESTplus toolkits (Jia et al., 2019). The first 10 volumes of each functional time series were discarded from analysis to allow for participant's stabilization and magnetic field equilibration. The remaining 130 volumes were corrected for the staggered order of slice acquisition that was used during echo-planar scanning. The correction ensures the data on each slice corresponds to the same point in time. The preprocessing also included regression of head motion parameters, realignment for head movement, and spatial normalization using T1 image unified segmentation to the Montreal Neurological Institute (MNI) space.

### 2.3. Dynamic causal modeling

We modeled the regions of interest (ROIs) as nodes in the EC networks. To include regions from DMN, SAN, and CEN networks, mPFC and bilateral PCC, dlPFC, ACC were chosen as regions of interest We summarized ROI activity by extracting time series at all voxels within a sphere having radius 8 *mm* around an associated MNI coordinate for the ROI. See Table 2 for MNI coordinates of each ROI. The coordinates were chosen based on previous investigations on bilateral PCC (Jeong et al., 2009), dlPFC (Gruber et al., 2010), ACC (Mannell et al., 2010), and mPFC (Maguire et al., 2010).

**Table 2:** Coordinates of ROI in MNI space.

**Regions**	**x**	**y**	**z**
L-dlPFC	–39	34	37
R-dlPFC	35	39	31
L-ACC	–5	39	20
R-ACC	6	33	16
L-PCC	–8	–49	38
R-PCC	8	–48	39
mPFC	0	53	–14

DCM is comprised of two models: the neuronal model and the observation model. For the resting state, the neuronal model tasks the form as follows:


(1) $$\begin{array}{l} \dot{x}(t)=Ax(t)+Cu+v \end{array}$$

In the equation above, *x* denotes the neuronal activity and *u* denotes stimulus. *v* denotes the random neuronal fluctuation that represents the state noise. Stimulus *u* is still included in the equation for the resting state scenario, but is usually set to zero in resting state models. The parameters θ = {*A, B*^*j*^, *C*} represent the intrinsic connectivity, extrinsic connectivity, and input, respectively.

The second model in DCM is the observation model. fMRI machines detect the activated brain regions by observing the changes in BOLD signal *y*. The generalized BOLD signal model was proposed by Stephan et al. (2007), in which both blood volume *v* and deoxyhemoglobin content *q* affect the observations:


(2) $$\begin{array}{l} y\left(t\right)\approx V_{0}\left[k_{1}\left(1-q\left(t\right)\right)+k_{2}\left(1-\frac{q\left(t\right)}{v\left(t\right)}\right)+k_{3}\left(1-v\left(t\right)\right)\right] \end{array}$$

where *V*_0_ is the resting venous blood volume fraction and *k*_1_, *k*_2_, *k*_3_ represent the coefficients associated with the machine's echo time and relaxation time. Changes in blood volume and deoxyhemoglobin level were caused by neuronal activities, and in turn, changed the observed BOLD signal responses. Thus the neuronal model and the observation model together captured the dynamics of the neural activity propagation.

We used PEB to infer the connectivity strengths for the four subject groups. In general, for PEB, a parameter vector is first sampled from a prior distribution. A random effect is added to the parameter vector for this subject. Then data are generated using the DCM and observation noise is added to model the observed response. The calculated connection strengths are accompanied by the likelihood of each connection. By setting the threshold for the likelihood, the most probable estimates are selected. Bayesian Model Reduction (BMR) was used to control the switching on and off of each connection (to calculate the likelihood with the connection's presence and absence) and estimate the parameter to infer the connectivity pattern. The connectivity strengths for each subject group were compared using analysis of variance (ANOVA). Bonferroni correction was applied for multiple comparisons. A linear regression analysis was also carried out to test if EC between regions could predict subjects' cognitive scores.

## 3. Results

### 3.1. Connectivity at individual level

**Figure 1:**
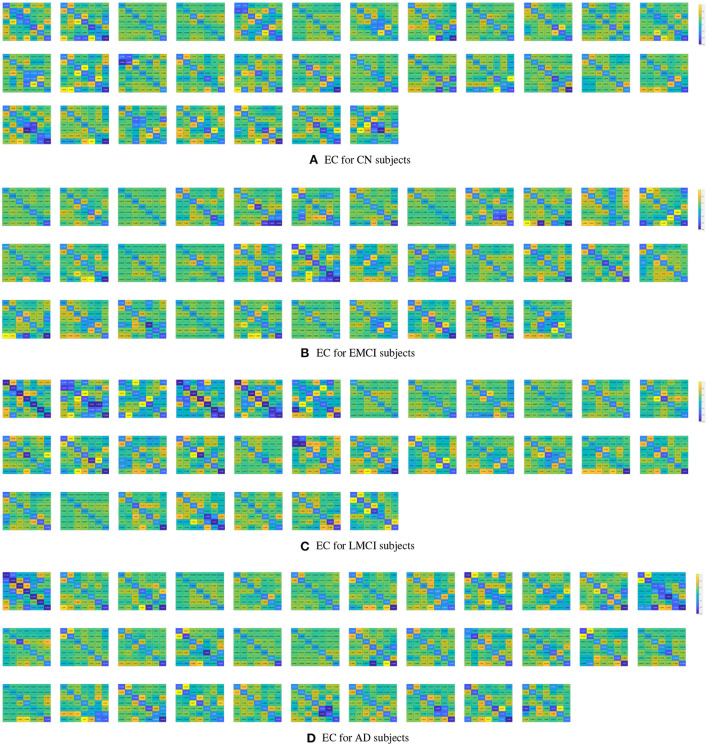
Subject-specific effective connectivity matrices following DCM of the resting-state fMRI for CN **(A)**, EMCI **(B)**, LMCI **(C)**, and AD **(D)** subjects. Each matrix element represents effective connectivity among 7 nodes in the following order: left PCC, right PCC, left ACC, right ACC, left dlPFC, right dlPFC, and mPFC. Directions of the connections are represented from columns to rows.

The estimated DCMs for the four subject groups are shown in Figure 1. The subject-specific matrices represent the intrinsic EC among the seven brain regions during the resting state.

### 3.2. Connectivity at group level

**Figure 2:**
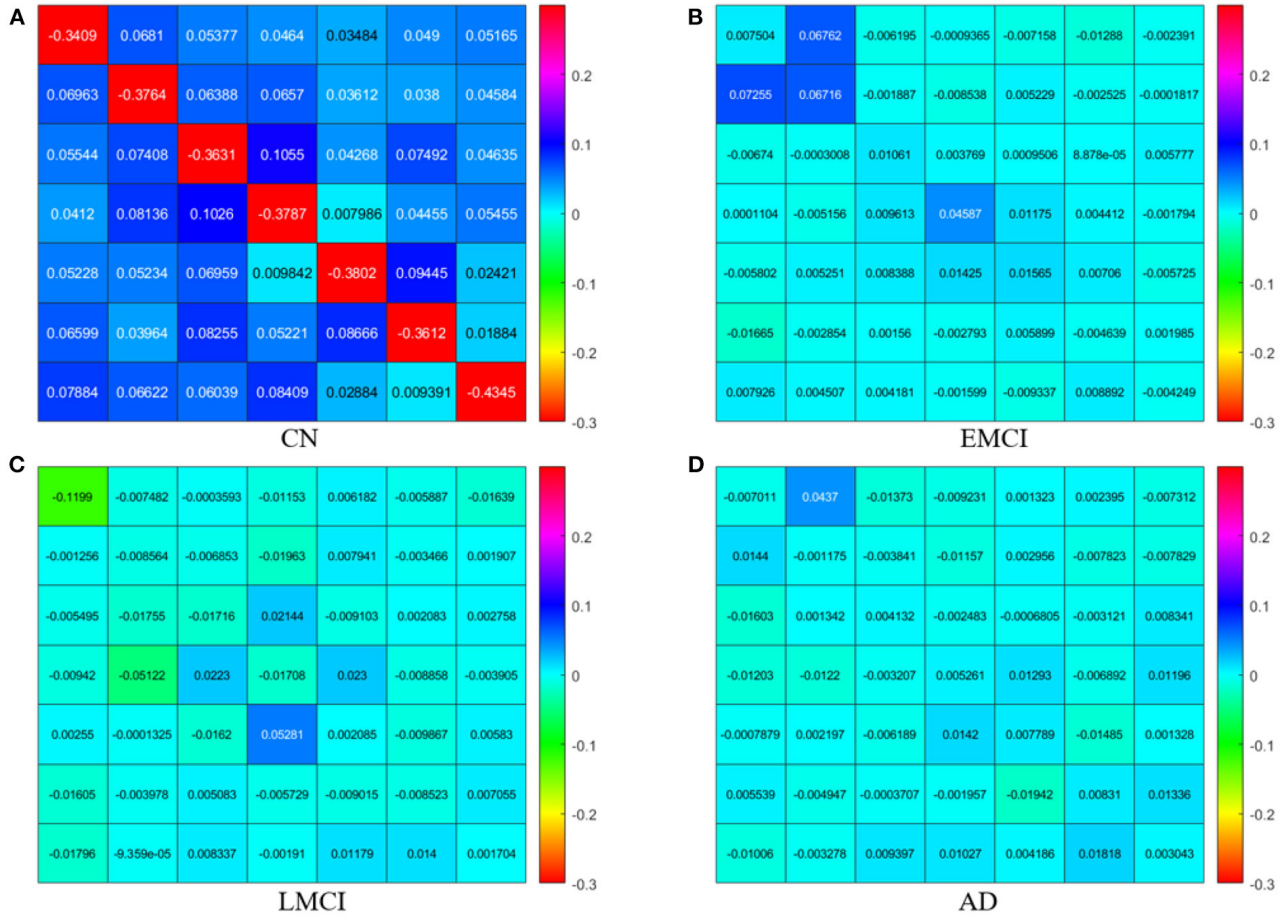
Effective connectivity for CN **(A)**, EMCI **(B)**, LMCI **(C)**, and AD **(D)** subject groups.

**Figure 3:**
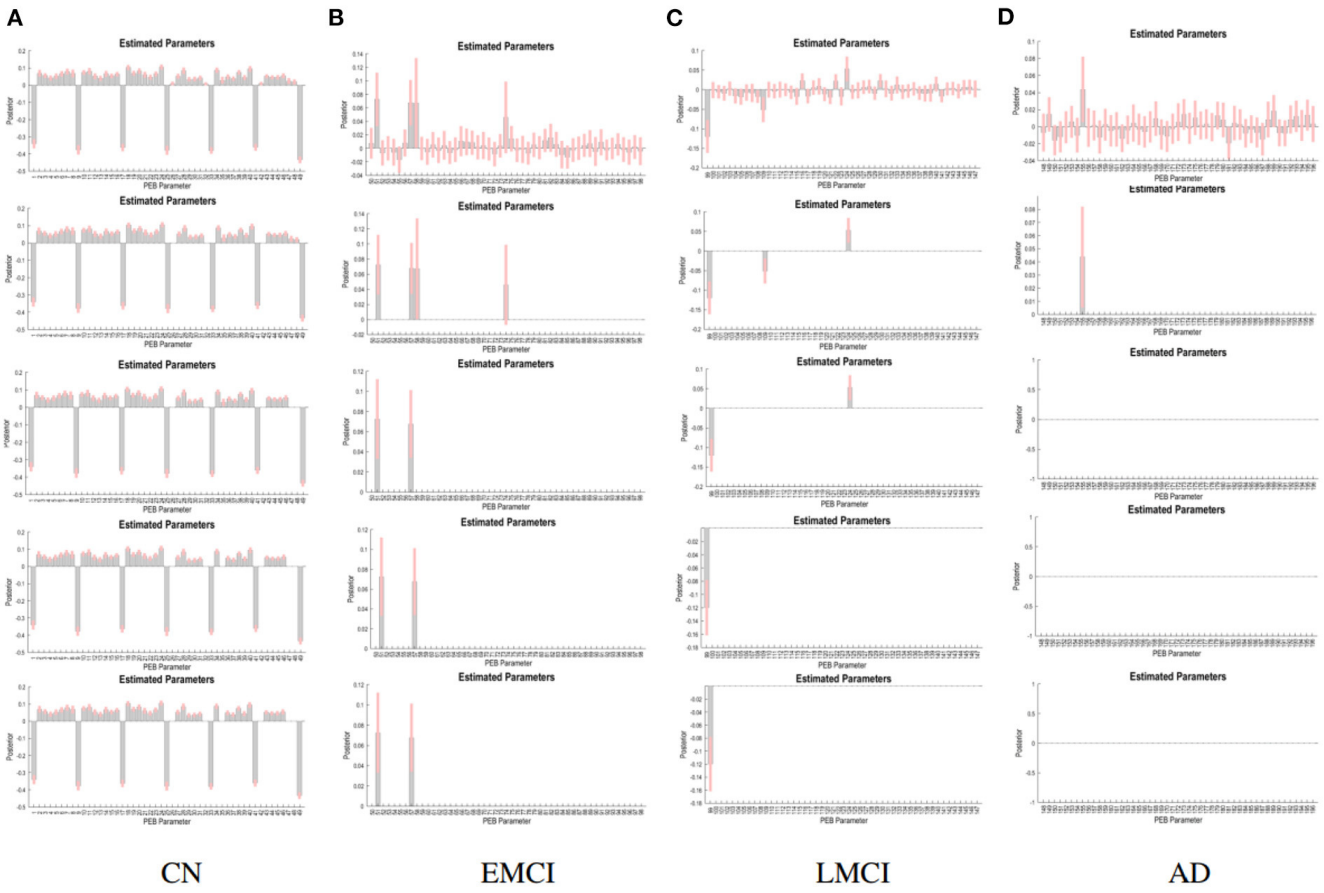
PEB estimates of connectivity for the four subject groups. Each row represents a threshold imposed on the evidence for each connection. The first row represents a no-threshold condition. The second, third, fourth, and fifth row represent free energy exceeding 0.5, 0.75, 0.95, and 0.99 conditions, respectively. CN **(A)**, EMCI **(B)**, LMCI **(C)**, and AD **(D)**.

The PEB process estimates group EC at five levels of confidence. At 0.5 level, all subject groups presented full connections (connections present among all ROI pairings). See Figure 2 for details of each subject group. However, the fully connected pattern only persisted for the CN group. Only two connections remained for the EMCI group after thresholding for 0.99 level. One connection (self connection L-PCC → L-PCC) remained for the LMCI group at 0.99 level. No connection was left after thresholding for the AD patient group. More information can be found in Figure 3.

**Figure 4:**
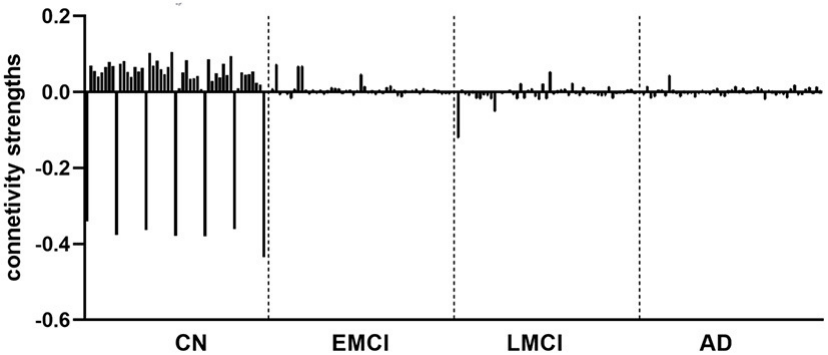
Effective connectivity for CN, EMCI, LMCI, and AD subject groups.

The group EC strengths were calculated following the calculation of connection probabilities. Posterior means and variances of all connection strengths for each subject group were presented in detail in Figure 4 and Tables 3–6. ANOVA of the effective connection strengths showed only connections L-PCC → L-PCC, R-PCC → R-ACC, and R-ACC → R-ACC were significantly different within subject groups. See details in Table 7.

**Table 3:** Connectivity strengths (means and variances) of CN subjects.

**From**	**To**	***M***	***Var***	**From**	**To**	***M***	***Var***
L-PCC	L-PCC	–3.409E-01	6.199E-04	L-dlPFC	L-PCC	3.484E-02	1.530E-04
	R-PCC	6.963E-02	3.062E-04		R-PCC	3.612E-02	1.464E-04
	L-ACC	5.544E-02	1.761E-04		L-ACC	4.268E-02	1.375E-04
	R-ACC	4.120E-02	2.040E-04		R-ACC	7.986E-03	1.581E-04
	L-dlPFC	5.228E-02	2.487E-04		L-dlPFC	-3.802E-01	4.622E-04
	R-dlPFC	6.599E-02	2.595E-04		R-dlPFC	8.666E-02	2.186E-04
	mPFC	7.884E-02	3.153E-04		mPFC	2.884E-02	2.817E-04
R-PCC	L-PCC	6.810E-02	2.755E-04	R-dlPFC	L-PCC	4.900E-02	1.725E-04
	R-PCC	–3.764E-01	6.527E-04		R-PCC	3.800E-02	1.521E-04
	L-ACC	7.408E-02	1.726E-04		L-ACC	7.492E-02	1.531E-04
	R-ACC	8.136E-02	2.196E-04		R-ACC	4.455E-02	1.638E-04
	L-dlPFC	5.234E-02	2.783E-04		L-dlPFC	9.445E-02	2.746E-04
	R-dlPFC	3.964E-02	2.076E-04		R-dlPFC	–3.612E-01	5.436E-04
	mPFC	6.622E-02	3.035E-04		mPFC	9.391E-03	2.791E-04
L-ACC	L-PCC	5.377E-02	1.601E-04	mPFC	L-PCC	5.165E-02	9.981E-05
	R-PCC	6.388E-02	1.628E-04		R-PCC	4.584E-02	1.029E-04
	L-ACC	-3.631E-01	5.412E-04		L-ACC	4.635E-02	1.305E-04
	R-ACC	1.026E-01	2.350E-04		R-ACC	5.455E-02	1.704E-04
	L-dlPFC	6.959E-02	2.084E-04		L-dlPFC	2.421E-02	1.640E-04
	R-dlPFC	8.255E-02	2.076E-04		R-dlPFC	1.884E-02	1.347E-04
	mPFC	6.039E-02	3.249E-04		mPFC	-4.345E-01	4.733E-04
R-ACC	L-PCC	4.640E-02	1.806E-04	R-ACC	L-dlPFC	9.842E-03	2.496E-04
	R-PCC	6.570E-02	1.899E-04		R-dlPFC	5.221E-02	1.962E-04
	L-ACC	1.055E-01	1.868E-04		mPFC	8.409E-02	3.808E-04
	R-ACC	–3.787E-01	6.092E-04				

**Table 4:** Connectivity strengths (means and variances) of EMCI patients.

**From**	**To**	***M***	***Var***	**From**	**To**	***M***	***Var***
L-PCC	L-PCC	7.504E-03	1.392E-03	L-dlPFC	L-PCC	–7.158E-03	3.091E-04
	R-PCC	7.255E-02	6.470E-04		R-PCC	5.229E-03	2.948E-04
	L-ACC	-6.740E-03	3.921E-04		L-ACC	9.506E-04	2.746E-04
	R-ACC	1.104E-04	4.442E-04		R-ACC	1.175E-02	3.170E-04
	L-dlPFC	–5.802E-03	5.360E-04		L-dlPFC	1.565E-02	9.202E-04
	R-dlPFC	–1.665E-02	5.609E-04		R-dlPFC	5.899E-03	4.342E-04
	mPFC	7.926E-03	6.711E-04		mPFC	–9.337E-03	5.716E-04
R-PCC	L-PCC	6.762E-02	5.799E-04	R-dlPFC	L-PCC	–1.288E-02	3.344E-04
	R-PCC	6.716E-02	1.344E-03		R-PCC	–2.525E-03	2.922E-04
	L-ACC	–3.008E-04	3.713E-04		L-ACC	8.878E-05	2.918E-04
	R-ACC	–5.156E-03	4.654E-04		R-ACC	4.412E-03	3.118E-04
	L-dlPFC	5.251E-03	5.813E-04		L-dlPFC	7.060E-03	5.344E-04
	R-dlPFC	–2.854E-03	4.369E-04		R-dlPFC	–4.639E-03	1.031E-03
	mPFC	4.507E-03	6.313E-04		mPFC	8.892E-03	5.481E-04
L-ACC	L-PCC	–6.195E-03	3.188E-04	mPFC	L-PCC	–2.391E-03	1.884E-04
	R-PCC	–1.887E-03	3.256E-04		R-PCC	–1.817E-04	1.953E-04
	L-ACC	1.061E-02	1.076E-03		L-ACC	5.777E-03	2.484E-04
	R-ACC	9.613E-03	4.618E-04		R-ACC	–1.794E-03	3.265E-04
	L-dlPFC	8.388E-03	4.118E-04		L-dlPFC	–5.725E-03	3.153E-04
	R-dlPFC	1.560E-03	4.094E-04		R-dlPFC	1.985E-03	2.557E-04
	mPFC	4.181E-03	6.446E-04		mPFC	–4.249E-03	8.931E-04
R-ACC	L-PCC	-9.365E-04	3.535E-04	R-ACC	L-dlPFC	1.425E-02	4.945E-04
	R-PCC	–8.538E-03	3.734E-04		R-dlPFC	–2.793E-03	3.781E-04
	L-ACC	3.769E-03	3.685E-04		mPFC	–1.599E-03	7.549E-04
	R-ACC	4.587E-02	1.121E-03				

**Table 5:** Connectivity strengths (means and variances) of LMCI patients.

**From**	**To**	***M***	***Var***	**From**	**To**	***M***	***Var***
L-PCC	L-PCC	–1.199E-01	1.015E-03	L-dlPFC	L-PCC	6.182E-03	2.909E-04
	R-PCC	–1.256E-03	5.652E-04		R-PCC	7.941E-03	2.758E-04
	L-ACC	–5.495E-03	3.014E-04		L-ACC	–9.103E-03	2.560E-04
	R-ACC	–9.420E-03	3.543E-04		R-ACC	2.300E-02	2.967E-04
	L-dlPFC	2.550E-03	4.434E-04		L-dlPFC	2.085E-03	8.191E-04
	R-dlPFC	–1.605E-02	4.667E-04		R-dlPFC	–9.015E-03	4.198E-04
	mPFC	–1.796E-02	5.707E-04		mPFC	1.179E-02	5.486E-04
R-PCC	L-PCC	–7.482E-03	5.010E-04	R-dlPFC	L-PCC	–5.887E-03	3.081E-04
	R-PCC	–8.564E-03	1.019E-03		R-PCC	–3.466E-03	2.682E-04
	L-ACC	–1.755E-02	2.905E-04		L-ACC	2.083E-03	2.688E-04
	R-ACC	–5.122E-02	3.840E-04		R-ACC	–8.858E-03	2.847E-04
	L-dlPFC	–1.325E-04	4.950E-04		L-dlPFC	–9.867E-03	5.166E-04
	R-dlPFC	–3.978E-03	3.545E-04		R-dlPFC	–8.523E-03	8.884E-04
	mPFC	–9.359E-05	5.348E-04		mPFC	1.400E-02	5.155E-04
L-ACC	L-PCC	–3.593E-04	2.845E-04	mPFC	L-PCC	–1.639E-02	1.856E-04
	R-PCC	–6.853E-03	2.953E-04		R-PCC	1.907E-03	1.919E-04
	L-ACC	–1.716E-02	9.317E-04		L-ACC	2.758E-03	2.480E-04
	R-ACC	2.230E-02	4.335E-04		R-ACC	–3.905E-03	3.266E-04
	L-dlPFC	–1.620E-02	3.768E-04		L-dlPFC	5.830E-03	3.145E-04
	R-dlPFC	5.083E-03	3.784E-04		R-dlPFC	7.055E-03	2.541E-04
	mPFC	8.337E-03	6.097E-04		mPFC	1.704E-03	8.557E-04
R-ACC	L-PCC	–1.153E-02	3.178E-04	R-ACC	L-dlPFC	5.281E-02	4.550E-04
	R-PCC	–1.963E-02	3.398E-04		R-dlPFC	–5.729E-03	3.422E-04
	L-ACC	2.144E-02	3.367E-04		mPFC	–1.910E-03	7.172E-04
	R-ACC	–1.708E-02	9.975E-04				

**Table 6:** Connectivity strengths (means and variances) of AD patients.

**From**	**To**	***M***	***Var***	**From**	**To**	***M***	***Var***
L-PCC	L-PCC	–7.011E-03	1.168E-03	L-dlPFC	L-PCC	1.323E-03	2.828E-04
	R-PCC	1.440E-02	5.774E-04		R-PCC	2.956E-03	2.704E-04
	L-ACC	–1.603E-02	3.334E-04		L-ACC	–6.805E-04	2.516E-04
	R-ACC	–1.203E-02	3.841E-04		R-ACC	1.293E-02	2.900E-04
	L-dlPFC	–7.879E-04	4.708E-04		L-dlPFC	7.789E-03	8.131E-04
	R-dlPFC	5.539E-03	4.926E-04		R-dlPFC	–1.942E-02	4.063E-04
	mPFC	–1.006E-02	6.036E-04		mPFC	4.186E-03	5.329E-04
R-PCC	L-PCC	4.370E-02	5.260E-04	R-dlPFC	L-PCC	2.395E-03	3.132E-04
	R-PCC	–1.175E-03	1.205E-03		R-PCC	–7.823E-03	2.732E-04
	L-ACC	1.342E-03	3.316E-04		L-ACC	–3.121E-03	2.745E-04
	R-ACC	–1.220E-02	4.189E-04		R-ACC	–6.892E-03	2.915E-04
	L-dlPFC	2.197E-03	5.355E-04		L-dlPFC	–1.485E-02	5.105E-04
	R-dlPFC	–4.947E-03	3.930E-04		R-dlPFC	8.310E-03	9.384E-04
	mPFC	–3.278E-03	5.842E-04		mPFC	1.818E-02	5.201E-04
L-ACC	L-PCC	–1.373E-02	2.948E-04	mPFC	L-PCC	–7.312E-03	1.835E-04
	R-PCC	–3.841E-03	3.021E-04		R-PCC	–7.829E-03	1.897E-04
	L-ACC	4.132E-03	1.005E-03		L-ACC	8.341E-03	2.437E-04
	R-ACC	–3.207E-03	4.381E-04		R-ACC	1.196E-02	3.169E-04
	L-dlPFC	–6.189E-03	3.858E-04		L-dlPFC	1.328E-03	3.076E-04
	R-dlPFC	–3.707E-04	3.839E-04		R-dlPFC	1.336E-02	2.490E-04
	mPFC	9.397E-03	6.201E-04		mPFC	3.043E-03	8.657E-04
R-ACC	L-PCC	-9.231E-03	3.321E-04	R-ACC	L-dlPFC	1.420E-02	4.658E-04
	R-PCC	–1.157E-02	3.478E-04		R-dlPFC	–1.957E-03	3.551E-04
	L-ACC	–2.483E-03	3.496E-04		mPFC	1.027E-02	7.185E-04
	R-ACC	5.261E-03	1.077E-03				

**Table 7:** ANOVA results for the effective connections.

**Connection**	**(I) group**	**(J) group**	**Mean difference (I-J)**	**Std. error**	**Sig**.
L-PCC → L-PCC	CN	EMCI	0.0002	0.0158	1.0000
		LMCI	0.0426	0.0159	0.0502
		AD	0.0275	0.0156	0.4792
	EMCI	CN	–0.0002	0.0158	1.0000
		LMCI	0.0424	0.0160	0.0552
		AD	0.0272	0.0157	0.5084
	LMCI	CN	–0.0426	0.0159	0.0502
		EMCI	–0.0424	0.0160	0.0552
		AD	–0.0152	0.0158	1.0000
	AD	CN	–0.0275	0.0156	0.4792
		EMCI	–0.0272	0.0157	0.5084
		LMCI	0.0152	0.0158	1.0000
**R-PCC** ** → R-ACC**	CN	EMCI	0.0252	0.0148	0.5469
		LMCI	0.0503	0.0149	0.0060
		AD	0.0268	0.0146	0.4113
	EMCI	CN	–0.0252	0.0148	0.5469
		LMCI	0.0251	0.0150	0.5887
		AD	0.0016	0.0147	1.0000
	LMCI	CN	–0.0503	0.0149	0.0060
		EMCI	–0.0251	0.0150	0.5887
		AD	–0.0235	0.0148	0.6941
	AD	CN	–0.0268	0.0146	0.4113
		EMCI	–0.0016	0.0147	1.0000
		LMCI	0.0235	0.0148	0.6941
R-ACC → R-ACC	CN	EMCI	–0.0102	0.0152	1.0000
		LMCI	0.0391	0.0154	0.0726
		AD	0.0080	0.0150	1.0000
	EMCI	CN	0.0102	0.0152	1.0000
		LMCI	0.0493	0.0155	0.0110
		AD	0.0182	0.0151	1.0000
	LMCI	CN	–0.0391	0.0154	0.0726
		EMCI	–0.0493	0.0155	0.0110
		AD	–0.0311	0.0152	0.2624
	AD	CN	–0.0080	0.0150	1.0000
		EMCI	–0.0182	0.0151	1.0000
		LMCI	0.0311	0.0152	0.2624

**Figure 5:**
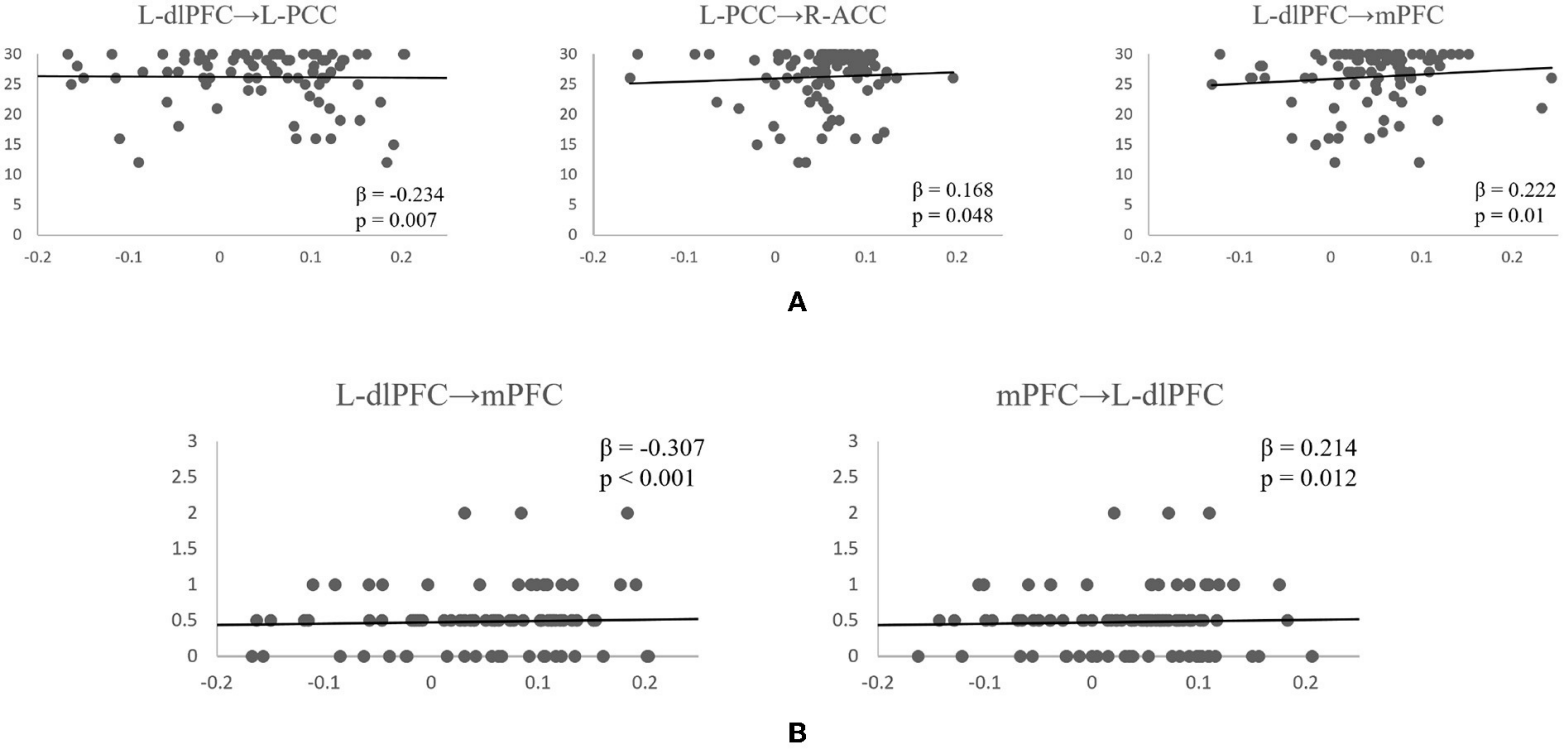
Scatterplots of significant effective connectivities vs. MMSE scores **(A)** and CDR scores **(B)**. Linear fit lines are shown. Both β and *p* values for each correlation are shown.

Following the PEB analysis, a linear regression analysis was carried out to test if resting-state connectivity between the brain regions could predict subjects' cognitive scores. For MMSE, the forward step-wise linear regression showed the EC between left dlPFC to left PCC was the first variable automatically entered into the step-wise regression (β = −0.234, *p* = 0.007), followed by connection between L-dlPFC to mPFC (β = 0.222, *p* = 0.01), and connection between L-PCC to R-ACC (β = 0.168, *p* = 0.048). Together, they explain 11.5% of variance in MMSE scores (*R*^2^ = 0.115, *F* = 5.434, *p* = 0.002). For CDR, both forward and backward connections between L-dlPFC and mPFC are predictive of the score (*R*^2^ = 0.121, *F* = 8.755, *p* < 0.001). Connection from L-dlPFC to mPFC negatively predicted the score (β = −0.307, *p* < 0.001), and connection from mPFC to L-dlPFC positively predicted the score (β = 0.214, *p* = 0.012). Scatter plots of effective connections vs. MMSE and CDR scores are shown in Figure 5.

## 4. Discussion

The DCM results revealed that the effective connection pattern and strength were different among the multiple stages of AD subjects. As expected, the EC analysis showed that the EC of EMCI, LMCI, and AD patients was reduced compared to that of CN subjects. The number of connections reduced from full connection in CN group to no connection in AD group. Statistical analysis revealed that for connection R-PCC → R-ACC, connection strength of CN is greater than that of LMCI. For connection R-ACC → R-ACC, connection strength of EMCI is greater than that of LMCI. In addition, for connection L-PCC → L-PCC, the connection strength of CN and EMCI is marginally greater than that of LMCI. Cognitive scores were also partially predicted by connectivity strengths of specific connections. MMSE scores were partially predicted by connection L-dlPFC → L-PCC, L-dlPFC → mPFC, and L-PCC → R-ACC. CDR scores were partially predicted by connection L-dlPFC → mPFC and mPFC → L-dlPFC.

The number of ECs was reported to dwindle steadily throughout the AD progression. Consistent with the results reported in this study, Wu et al. (2011) found that connections between mPFC and PCC were all lost in AD patients compared to CN subjects. Rytsar et al. (2011) also reported that AD was associated with significantly weakened effective connections. Although their study was focused on the visual cortex, it nonetheless provides evidence that loss of EC is common in AD progression. Neufang et al. (2011) related the connectivity parameters to subjects' gray matter volume and found gray matter volume at the right middle frontal gyrus significantly correlated with connectivity strengths. They concluded that the reduction of EC contributes to brain control impairments in AD patients.

Although not enough EC analyzes have been carried out for AD, evidence from FC studies also showed the degenerative effect of disease progression on the communications between brain regions. For example, Binnewijzend et al. (2012) investigated regional FC in DMN of CN, MCI, and AD patients. Although the statistical difference was only observed between AD and MCI patients, they reported numerical decreases of FC from CN to MCI to AD. A meta-review of past MCI studies by Xu et al. (2020) suggested increased FCs in MCI patients were located in the precentral gyrus and middle frontal gyrus, and decreased FCs were located in the middle frontal gyrus, cingulate gyrus, and superior frontal gyrus. They concluded that the effect of AD progression is presented in the interactive neural networks and that dysfunctional connectivity may reflect the gradual decline from MCI to AD. The results from our study agree with the notion that disruption in connectivities can be detected in early stage of AD, as early as EMCI stage.

Only a handful of studies have investigated the EC of AD patients. Among them, even fewer studies looked into the details of the connection strengths and their relationships with cognitive scores. Chand et al. (2017b) studied the interaction of DMN, CEN, and SAN and showed that disruption in SAN correlated significantly with lower cognitive performance. In the present study, we found the interactions between DMN and CEN significantly affected cognitive performance. Connections between L-dlPFC, PCC, and mPFC partially predict both the MMSE and CDR scores. ACC anchored in SAN did not demonstrate a great effect on cognitive scores. However, it is worth noting that connections involving ACC were sensitive to the disease progression. Lower connection strengths were observed for later-stage subjects in connections involving ACC.

Considering the relationship of EC with FC, Neufang et al. (2011) found that two connectivities are significantly related in healthy subjects. Yet they pointed out this kind of similarity is disrupted in AD patients. FC strengths are not good indicators of EC strengths in AD patients. In fronto-cingulo-parietal connections, subjects with AD showed significant differences between the measures. Past studies associated such differences with age rather than AD (Raji et al., 2009), yet the study on EC among patients within different AD stages suggested otherwise. The Cingulate is an important region as well as a vital part of fronto-cingulo connections. The strengths of connections involving cingulate are predictive of cognitive scores as shown in this study. This could be due to disconnections among the networks, or it could be the result of the impaired integration of the cingulate cortex itself. Further studies are still needed to clarify the exact role of cingulate cortex and its connections in AD progression. Overall, the results highlighted the important progressively disrupting effect of AD on DMN, CEN, and SAN. We believe such an effect could be valuable for the classification and prediction of AD stages. The altered connectivity strengths combined with other symptoms and biological information of the patients could be used as classification features in patient diagnoses.

Due to the relatively small sample size of the current study, further investigation of AD progression is still needed to draw a conclusion of the generative effect of the progression on the connectivity strengths. We did not include other complications (e.g., depression, Parkinson's disease) in our current study, but as these are common diseases among AD patients, further studies are still needed to look into the combined effect of multiple neurological disorders on brain connectivities. Further studies should also include brain signals captured under other experimental conditions in addition to the resting state and investigate the intrinsic brain dynamics.

## 5. Conclusion

The results of this study showed the potential of EC as a biomarker in predicting and classifying AD progression. Reduced ECs were reported in later stages of AD progression compared to CN subjects. The directional information revealed exclusively with EC using DCM has contributed, and may further contribute, to our understanding of the progression of Alzheimer's disease.

## Alzheimer's Disease Neuroimaging Initiative

Data collection and sharing for this project was funded by the Alzheimer's Disease Neuroimaging Initiative (ADNI) (National Institutes of Health Grant U01 AG024904) and DOD ADNI (Department of Defense award number W81XWH-12-2-0012). ADNI is funded by the National Institute on Aging, the National Institute of Biomedical Imaging and Bioengineering, and through generous contributions from the following: AbbVie, Alzheimer's Association; Alzheimer's Drug Discovery Foundation; Araclon Biotech; BioClinica, Inc.; Biogen; Bristol-Myers Squibb Company; CereSpir, Inc.; Cogstate; Eisai Inc.; Elan Pharmaceuticals, Inc.; Eli Lilly and Company; EuroImmun; F. Hoffmann-La Roche Ltd and its affiliated company Genentech, Inc.; Fujirebio; GE Healthcare; IXICO Ltd.; Janssen Alzheimer Immunotherapy Research & Development, LLC.; Johnson & Johnson Pharmaceutical Research & Development LLC.; Lumosity; Lundbeck; Merck & Co., Inc.; Meso Scale Diagnostics, LLC.; NeuroRx Research; Neurotrack Technologies; Novartis Pharmaceuticals Corporation; Pfizer Inc.; Piramal Imaging; Servier; Takeda Pharmaceutical Company; and Transition Therapeutics. The Canadian Institutes of Health Research is providing funds to support ADNI clinical sites in Canada. Private sector contributions are facilitated by the Foundation for the National Institutes of Health (www.fnih.org). The grantee organization is the Northern California Institute for Research and Education, and the study is coordinated by the Alzheimer's Therapeutic Research Institute at the University of Southern California. ADNI data are disseminated by the Laboratory for Neuro Imaging at the University of Southern California.

## Data availability statement

The original contributions presented in the study are included in the article/supplementary material, further inquiries can be directed to the corresponding author.

## Author contributions

JH, J-YJ, and CN contributed to the conception and design of the study and to the interpretation of the findings of the study. JH selected and analyzed the data. JH and CN drafted the manuscript. All authors contributed to the article and approved the submitted version.
